# Retrograde Signaling: Understanding the Communication between Organelles

**DOI:** 10.3390/ijms21176173

**Published:** 2020-08-26

**Authors:** Jakub Mielecki, Piotr Gawroński, Stanisław Karpiński

**Affiliations:** Department of Plant Genetics, Breeding and Biotechnology, Institute of Biology, Warsaw University of Life Sciences, 02-787 Warsaw, Poland; jakub_mielecki@sggw.edu.pl (J.M.); piotr_gawronski@sggw.edu.pl (P.G.)

**Keywords:** retrograde signaling, biogenic control, operational control, stress response, cell death

## Abstract

Understanding how cell organelles and compartments communicate with each other has always been an important field of knowledge widely explored by many researchers. However, despite years of investigations, one point—and perhaps the only point that many agree on—is that our knowledge about cellular-signaling pathways still requires expanding. Chloroplasts and mitochondria (because of their primary functions in energy conversion) are important cellular sensors of environmental fluctuations and feedback they provide back to the nucleus is important for acclimatory responses. Under stressful conditions, it is important to manage cellular resources more efficiently in order to maintain a proper balance between development, growth and stress responses. For example, it can be achieved through regulation of nuclear and organellar gene expression. If plants are unable to adapt to stressful conditions, they will be unable to efficiently produce energy for growth and development—and ultimately die. In this review, we show the importance of retrograde signaling in stress responses, including the induction of cell death and in organelle biogenesis. The complexity of these pathways demonstrates how challenging it is to expand the existing knowledge. However, understanding this sophisticated communication may be important to develop new strategies of how to improve adaptability of plants in rapidly changing environments.

## 1. Introduction

Oxygenic photosynthesis is an ancient process that likely evolved over 3.7 billion years ago in free-living bacteria. According to the endosymbiosis theory, the ancestor of the current alphaproteobacteria from which mitochondria are derived were incorporated into prokaryotic Archaean cells. Some of these newly formed eukaryotic cells underwent another endosymbiosis event, incorporating a photosynthetically active ancestor of cyanobacteria into their cells. These two events—followed by a long period of evolution—resulted in the emergence of modern eukaryotic plant cells [1]. During the evolution of eukaryotic plant cells, the genomes of the original endosymbionts evolved and rearranged in such way that many genes were transferred from organellar genomes to nucleus. This process was aimed at securing endosymbionts in eukaryotic cell and simplifying metabolic pathways to allow eukaryotic cells to manage their resources in a more efficient manner. However, these genetic rearmaments during the evolution of the modern plant cell also required the evolution of a communication between the prokaryotic precursors of organelles and the eukaryotic nucleus.

During evolution, the majority of genes encoded by the genome of free-living cyanobacteria were transferred to the nuclear genome of the newly formed symbiotic cell [2,3]. Around 18% of the *Arabidopsis thaliana* nuclear genes are derived from a plastid ancestor. Surprisingly, the majority of proteins encoded by those genes are not transferred back to the chloroplasts. Even in nonphotosynthetic organisms like *Plasmodium* or *Trypanosoma*—which evolved from red algae—we still find some functional genes of cyanobacteria origin [4]. This demonstrate that the benefits from endosymbiosis can be various and the evolution of eukaryotes took many different paths in order to gain advantage over prokaryotic cells. We cannot clearly distinguish which pathway was more beneficial, but it is obvious that the emergence of the eukaryotic cell led to the possibility for the evolution of plant, fungi and animal modern cells, which was a major breakthrough in evolution of oxygenic life on earth. To make this happen, the original symbionts had to develop a highly coordinated two—or three-way communication system. It is obvious that the expression of many genes encoded by the nuclear genome depends on organellar signals, in a mechanism called retrograde signaling. In this review, we present role of the signals derived from dysfunctional organelles such as chloroplasts, mitochondria and peroxisomes in regulation of nuclear gene expression (NGE). The proper folding and assembly of many plastid protein complexes (e.g., photosystems) requires the highly coordinated coupled expression of photosynthesis-associated nuclear genes (PhANGs) and photosynthesis-associated plastid genes (PhAPGs). On the other hand, organellar gene expression (OGE) is controlled by nuclear-encoded factors; this kind of regulation is called anterograde signaling. Disturbances in retrograde—as well as anterograde—signaling may lead to their uncoupled expression, which has harmful effects on plant cells by impairing the proper functioning of chloroplasts [5]. Under photooxidative stress, chloroplasts are not able to carry out the biosynthesis of carbohydrates efficiently, which limits the energy supply of cells. Instead, excess excitation energy (EEE) results in a rapid foliar temperature increase due to nonphotochemical quenching (NPQ) and formation of reactive oxygen species (ROS) which—apart from their destructive capabilities—are also able to act as signaling molecules, allowing plants to adapt to suboptimal environmental conditions. The inhibition of mitochondrial electron transport and the tricarboxylic acid cycle requires retrograde induction of the alternative oxidase pathways in order to reduce oxygen and maintain energy production. Alternative respiratory pathways have a lower ATP yield than the main pathway. However, in stressful conditions, it may be the only way to produce energy. Severe oxidative stress can surpass the abilities of the antioxidative system, and ultimately results in the induction of programmed cell death (PCD). Plants induce PCD in order to survive in extreme environmental conditions, or to prevent spread of pathogens which eventually could lead to death of the whole plant organism. Retrograde signals originated from peroxisomes are connected with the inhibition of catalases. Recently, the role of peroxisome-derived H_2_O_2_ in the induction of PCD was established; we will discuss this later in this review. On the other hand—apart from discussing the role of each mentioned organelle in retrograde signaling—we also present several putative signal integrators, indicating that under some conditions, changes in the NGE may require a coordinated signal from more than one type of organelle.

Crosstalk between the nucleus and other organelles is crucial, not only for their development, but also to trigger stress responses or to acclimate to a constantly changing environment. Understanding of those complex signaling pathways may allow us to modify them in the future in order to improve crop productivity or to respond to several combined abiotic and biotic stresses [6,7,8,9].

Along with climate change and the overheating of the Earth which we are experiencing nowadays, extreme conditions such as water deficit, extreme temperatures, storms, deforestation, desert expansion and high soil salinity occurs more often [10,11]. The Pace of those changes raises at least a few questions: Will plants be able to cope with such rapid climate change? What about diseases and pests that will migrate to more favorable habitats? This is currently happening, for example in the pandemic wheat disease—stem rust (TTTTF)—which is migrating from Africa to Europe [12]. This is why we should think about these challenges more carefully in coming years. If not, we will be not prepared for what may come [13]. One strategy to resolve this problem is to enhance natural plant defense and acclimation mechanisms in crops with genes from their wild ancestors. Especially helpful in this case may be some knowledge about retrograde-signaling pathways. However, acclimation to stressful conditions requires energetic investment which could be utilized for growth and development. Finding the proper balance between those two processes may be crucial [14].

Even widely described, retrograde-signaling pathways still has some unknowns (mostly how signals are transduced from organelles to nucleus). Recently, some scientists working in this field question that even genes that has been considered to be key parts of some retrograde-signaling pathways for last few years. In this review, we try to describe different classes of retrograde signals, already known pathways related to organelles biogenesis or stress response and also discuss all the controversy that have recently arisen around some of the putative retrograde candidates.

## 2. Plurality of Putative Retrograde Signals

Retrograde signals can be classified according to the nature of the signal. We can distinguish few major types of the signals which are transduced by different biomolecules such as: RNA, ROS, proteins and other metabolites. In pioneering work of chloroplast-to-nucleus signaling, it was proposed that RNA from plastids can regulate protein synthesis in cytoplasm [15]. Indeed, recently was identified link between retrograde signaling and RNA editing in chloroplasts [16]. Noteworthy, recent studies show that RNA metabolism processes such as alternative splicing and micro RNA synthesis are not only affected by retrograde signaling but can also trigger it as well. One hypothesis indicates that it could be achieved through alterations in RNA editing levels for transcripts encoding subunits of RNA polymerase such as *rpoC1* and *rpoB* [17,18,19,20]. Even without evidence of RNA being signaling molecule for retrograde signaling itself, it is clear that RNA metabolism (both nuclear and plastid) plays major role in transduction of retrograde signals.

**Figure 1:**
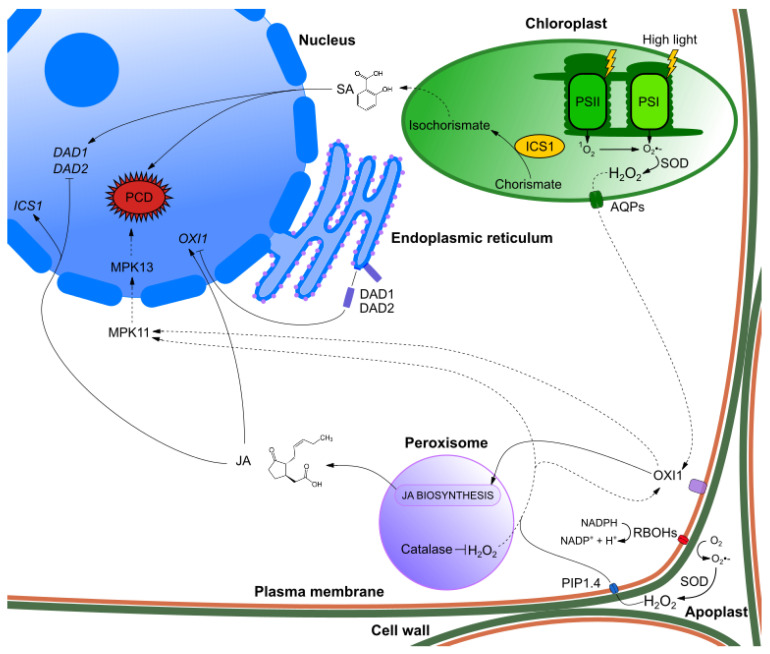
Role of oxidative signal inducible 1 (OXI1) in retrograde signaling in mature mesophyll cells. OXI1 in response to reactive oxygen species (ROS) induces biosynthesis of jasmonic acid (JA) and induces programmed cell death (PCD) through MPK11/MPK13-mediated pathway. JA induces expression of *ICS1*. Crosstalk between SA and JA along with endoplasmic reticulum (ER)-associated proteins DEFENDER AGAINST CELL DEATH (DAD1)/DAD2 regulates expression of *OXI1*. Dotted lines represent pathways which lack experimental evidence or exact nature of indicated regulation is still unknown.

ROS are products of several metabolic pathways; it was described that their accumulation can affect NGE [21]. ROS can be produced in apoplast, chloroplasts, mitochondria, glyoxysomes, peroxisomes, endoplasmic reticulum (ER) and cytosol [22]. Photosynthesis and photorespiration are well known metabolic processes that generates ROS. NADPH oxidases (NOXs) activity results in ROS formation in apoplast. Respiratory burst oxidase homologs (RBOHs) are plant NADPH oxidases located in the plasma membrane. Apoplastic ROS are scavenged in the same way as it happens intracellular, but by a specific extracellular isoforms of antioxidative enzymes [23,24]. H_2_O_2_ formed in apoplast can be transported into the cells via aquaporins (AQPs) (Figure 1). Plasma membrane intrinsic protein 1.4 (AtPIP1.4) was described as the AQP that can facility intracellular H_2_O_2_ transport through plasma membrane from apoplast [25]. This mechanism may allow apoplastic communication between plant cells and allow induction of systemic responses. Redox state of the apoplast is important in acclimation of photosynthesis to variable light intensity [26]. To further support this hypothesis recently apoplastic H_2_O_2_ sensor—HPCA1—was identified [27]. Cells developed ROS scavenging mechanisms to maintain homeostasis, it is achieved mainly by enzymatic and nonenzymatic compounds [28]. Glutathione (GSH) and especially ascorbate (AsA) are main nonenzymatic antioxidants in plants [29]. However, those nonenzymatic compounds depend on their recycling by specific set of enzymes. For example, DEHYDROASCORBATE REDUCTASE2 (DHER2) is an important enzyme taking part in ascorbate recycling [30]. There are also other nonenzymatic antioxidative compounds such as phenolic compounds, carotenoids, flavonoids, tocopherols and alkaloids [31]. Mentioned compounds play role in regulating redox state of plant cells. Under abiotic or biotic stress ROS production surpass abilities of antioxidative systems eventually changing redox state of chloroplasts which is considered to be important in triggering retrograde signaling. Because some of ROS like singlet oxygen (^1^O_2_) have short lifespan it is clear that it rather takes part as one of the signaling molecules than migrate from plastids to nucleus itself to modulate NGE. It is still unclear how ROS escape chloroplasts. However, in cytoplasm there are several redox sensitive proteins such as Mitogen-activated protein kinases (MAPKs) which can trigger signal cascades that eventually lead to changes in NGE [32]. ROS stability, apart from their short half-time is also linked with their reactivity. More reactive species like hydroxyl radical (OH●) are chemically unstable and they can only oxidize compounds in their close vicinity. In contrast to hydroxyl radical, H_2_O_2_ is the most stable ROS. However, short-lived ROS like superoxide anion radical (O_2_•^−^) generated by photosystem I (PSI) can be dismutated to H_2_O_2_ enzymatically by superoxide dismutase (SOD) or spontaneously [33]. It was reported that hydrogen peroxide can oxidase Calvin cycle enzymes containing thiol group [34]. Oxidation of cysteine thiol group to sulfenic acid (Cys–SOH) is well established reversible post translational modification that regulates protein activity. It depends on the redox state of cell. Sulfenylation of important antioxidant enzyme DHER2 prevents it from irreversible overoxidation [30]. Endosymbiosis events also contributed to evolution of redox sensitive proteins containing cysteine thiols in order to sense and regulate redox state of cell [35]. Many hypotheses about role of H_2_O_2_ in plant signaling under light illumination were thoughtfully discussed in literature [36,37]. H_2_O_2_ derived from photosynthesis is also able to diffuse out of the chloroplasts during illumination [32,38,39]. However, authors suggested that it cannot pass chloroplast membrane by simple diffusion. It is more likely that it is transported out of chloroplasts through AQPs [40]. Some researchers hypothesize that it may be delivered from chloroplast to nucleus via stromules [41]. They are tubules filled with stroma formed by all plastid types discovered in vascular plants and it was described that they can emanate after exposure to ROS [42]. Recently, research group of Phillip Mullineaux suggested that photosynthesis derived H_2_O_2_ from chloroplasts associated nearby nuclei may affect NGE omitting transport by cytosol [43].

Hydrogen peroxide in chloroplasts is detoxified by peroxiredoxin (PrxR), but mostly by ascorbate–glutathione cycle in which ascorbate peroxidase (APX) is key enzyme [44,45]. There are different enzymes that can also scavenge ROS, including catalase (CAT), superoxide dismutase (SOD) and glutathione peroxidase (GPX). Because of their broad substrate specificity usually each ROS can be utilized by at least two different enzymes [46,47]. It is also worth mentioning that each cell compartment has specific protein isoforms and contain more than one type of antioxidative enzymes. For example, we can distinguish APX isoenzymes located in stromal (sAPX) and thylakoid (tAPX) membrane of chloroplasts (in mature mesophyll cells), cytosol APX (cAPX) and microbody APX (mAPX) bound to membrane of peroxisome and glyoxysome [48]. APX change their activity under high light illumination [49,50,51]. *APXs* expression is regulated by redox status of plastoquinone pool [50]. Existence of several APX isoenzymes allow plant cells to regulate ROS content in each compartment independently [52]. Cells may purposely not scavenge all ROS in efficient manner and in every cell compartment so the signal can be transduced and proper response for high light intensity induced. It is still unclear if response to H_2_O_2_ depends on in which organelle it was produced or rather signal is integrated regardless of the source [53]. Signaling pathways originating from peroxisomes were often discovered analyzing catalase mutants (*cat*) which accumulate H_2_O_2_ inside peroxisomes in differentiated cells. Its accumulation lead to the induction of pathogenesis related genes and ultimately to the cell death [54,55]. *cat* mutants differentially express many nuclear encoded genes compared to the wild-type plants under high light intensity stress [56]. Transcriptome analysis of *cat2* mutant shows higher expression of many genes related to protein repair [57]. More severe changes are observed in transcriptome of *cat1 cat2 cat3* triple mutant exhibiting deregulation in expression pattern of several receptor-like kinases and transcription factors (TFs) [58]. Among those genes *oxidative signal inducible 1* (*OXI1*) encoding serine/threonine kinase, *MPK11* and *MPK13* were especially interesting considering redox sensitivity of some MAPKs. OXI1 belongs to AGC family of plasma membrane bound kinases [59]. OXI1 was described as activator of MPK3 and MPK6 in response to H_2_O_2_ and pathogens [60]. However, no changes in expression pattern of those kinases were observed in triple catalase mutant [58]. This may suggest existence of separate H_2_O_2_ related signaling pathways for peroxisomes and chloroplasts. H_2_O_2_ accumulation in peroxisomes promote transduction of the signals through OXI1/MPK11/MPK13 (Figure 1). To support this statement, it was observed that *MPK3* and *MPK6* expression levels are not affected in *OXI1* overexpressing lines [61]. Recently, two PCD inhibitors: DEFENDER AGAINST CELL DEATH (DAD1) and its homolog DAD2 were described as regulators of OXI1 induced cell death in response to high light stress [61]. *DAD1* and *DAD2* overexpression lines exhibit lower expression of *OXI1* than wild-type and *OXI1* is upregulated in *dad1* and *dad2* mutants. This suggest antagonistic role of DAD1/DAD2 and OXI1. Induction of OXI1 dependent cell death is coregulated by two plant hormones salicylic acid (SA) and jasmonic acid (JA). SA apart from its role in pathogen defense signaling also regulates redox homeostasis and light acclimation [62]. Wild-type plants treated with JA exhibit increased expression of *OXI1* and decreased expression of *DAD1* and *DAD2*. *oxi1* mutants have lower content of JA than wild-type plants and high light treatment does not induce JA accumulation like it does in the wild-type. This suggest that OXI1 regulates JA biosynthesis in peroxisomes. In contrast, treatment with SA induces *DAD1* and *DAD2* expression while *OXI1* is expressed at same level. Treatment with JA induce expression of SA biosynthesis genes: *ENHANCED DISEASE SUSCEPTIBILITY1* (*EDS1*) and *ISOCHORISMATE SYNTHASE1* (*ICS1*) in the wild-type which leads to increased isochorismate biosynthesis in chloroplasts. ICS1 converts chorismate to isochorismate which is a precursor for SA biosynthesis in cytosol. In contrast treatment with SA does not affect expression of JA biosynthesis genes (*AOS* and *OPR3*) in the wild-type [61]. Regulation of PCD by OXI1 in response to highlight and H_2_O_2_ accumulation in peroxisomes are shown in Figure 1.

**Figure 2:**
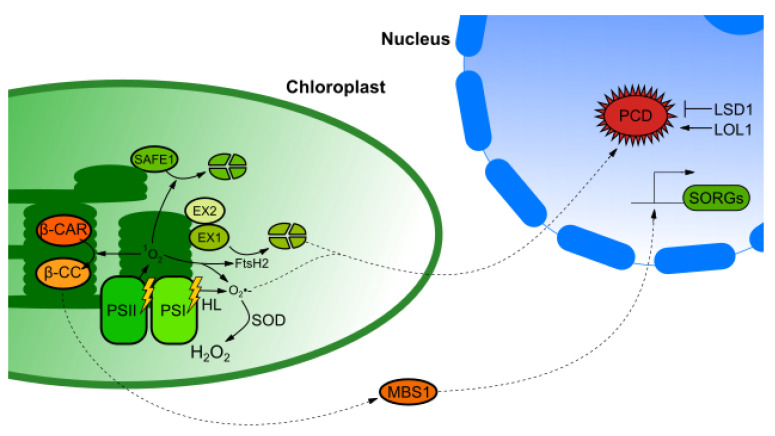
Retrograde-signaling pathways mediated by singlet oxygen in mature mesophyll cell. High light stress induces ROS production in photosystem I (PSI) and PSII. Depending on its concentration singlet oxygen induce different pathways. At low concentrations it promotes degradation of EXECUTER1 (EX1) by FtsH2 protease and induce EX1-mediated PCD. It is negatively regulated by LESION STIMULATING DISEASE1 (LSD1) and positively by its homolog LOL1. SAFEGUARD1 (SAFE1) similarly, to EX1 protects grana margins from oxidative damage by singlet oxygen. At high concentration singlet oxygen oxidizes β-carotene to β-cyclocitral and induces expression of many singlet oxygen related genes (SORGs) through METHYLENE BLUE SENSITIVITY 1 (MBS1). Dotted lines represent pathways which lack experimental evidence or exact nature of indicated regulation is still unknown.

Another interesting aspect in singlet-oxygen–mediated signaling is the executer pathway. EXECUTER1 (EX1) and EXECUTER2 (EX2) are proteins associated with thylakoid membranes and were identified using *fluorescent* (*flu*) mutant suppressor screen [63,64]. Seedling of *Arabidopsis thaliana flu* mutant in the dark accumulate protochlorophyllide which is an intermediate in the biosynthesis of chlorophyll *a*. Transitioning mutants to light results in burst production of singlet oxygen which affect NGE and eventually leads to the inhibition of growth, chlorosis and ultimately cell death [65]. EX1 and EX2 are part of singlet-oxygen-dependent retrograde signaling under low light conditions [63]. In excess light conditions retrograde signals are transduced independently of EX1 and EX2 through generation of β-cyclocitral [66,67]. EX1 is localized to the grana margins in chloroplasts. Chlorophyll synthesis, disassemble and reassemble of damaged PSII take place in a close vicinity of EX1. Singlet-oxygen–mediated retrograde signaling depends on degradation of EX1 protein by ATP-dependent zinc metalloprotease FtsH. The FtsH also catalyzes cleavage of D1 protein in the reaction center of the damaged PSII. Based on that, it is suggested that EX1 dependent signaling is connected with repair of PSII [68]. EX1 degradation depends on oxidation of tryptophan at position 643 by singlet oxygen. The substitution of this amino acid with leucine or alanine (which also are singlet oxygen sensitive amino acids) inhibits EX1 degradation by FtsH2 [69]. Recently, another novel singlet oxygen induced retrograde singling pathway was discovered by ethyl methanesulfonate (EMS) mutagenization in *flu ex1* double mutant. SAFEGUARD1 (SAFE1) is localized in the stroma of chloroplasts and is degraded by the release of singlet oxygen. Plants lacking functional SAFE1 protein were more susceptible to singlet-oxygen-induced damage of thylakoids grana margins [70]. Singlet-oxygen-mediated retrograde-signaling pathways are demonstrated in Figure 2.

Retrograde signals can also be carried out by different classes of proteins in which transcription factors are worth mentioning. There are few well described proteins such as Whirly1, Plant homeodomain transcription factor with transmembrane domains (PTM) or ABSCISIC ACID-INSENSITIVE4 (ABI4) [71,72,73], but we will describe those widely in other section of this review.

One of most interesting and well known retrograde-signaling pathway is connected with tetrapyrrole biosynthesis. Under norflurazon (NF) treatment—which inhibits biosynthesis of carotenoids—several nuclear genes are downregulated [73,74]. Almost three decades ago research group of Joanne Chory identified several *genomes uncoupled* (*gun*) mutants under NF treatment [75]. *Arabidopsis thaliana* mutants exhibiting *gun* phenotype are not able to downregulate PhANGs (e.g., *LIGHT HARVESTING CHLOROPHYLL A/B BINDING PROTEIN1.2* (*LHCB1.2*)) like wild type plants after chloroplast damage caused by NF treatment [76]. Thus, it was concluded that GUNs are part of retrograde-signaling pathway regulating expression of PhANGs. All *GUNs* except *GUN1* encode proteins involved in tetrapyrrole biosynthesis pathway (TBP) in chloroplasts. GUN1 is pentatricopeptide repeat (PPR) protein localized in chloroplasts containing a small MutS related (SMR) domain. Analysis of *gun2-5* mutants initially led to conclusion that Mg–protoporphyrin (MgProto) is a retrograde metabolite able to move between the nucleus and chloroplasts [77]. However, further studies did not support this hypothesis because no correlation was found between level of MgProto and *LHCB1.2* expression [78]. In contrary some researchers suggested that this may be due to difficulties with identification of tetrapyrroles by HPLC while their content is low [79]. Recent study confirmed accumulation of MgProto in first two days after newly germinated seedlings were treated with NF. The accumulation of MgProto was also correlated with repression of *LHCB1.2* expression suggesting that accumulation of MgProto is a retrograde signal [80].

The identification of *gun6* mutant exhibiting higher activity of ferrochelatase 1 (FC1) suggested that heme synthetized by FC1 may be precursor or retrograde signal itself [81]. Overexpression of *FC1* targeted to chloroplasts rescued nuclear gene expression after NF treatment and increased expression levels of *CA1*, *LHCB2.1* and *GUN4* even without this treatment. However, targeting FC1 to mitochondria did not affect NGE [82]. These results further support role of heme synthetized by FC1 in the chloroplast-to-nucleus retrograde signaling. On the other hand, a link between ROS and tetrapyrroles pathway may be a GUN4 protein. GUN4 and Protoporphyrin IX form ^1^O_2_ generating complex which can initiate retrograde signaling [83].

**Figure 3:**
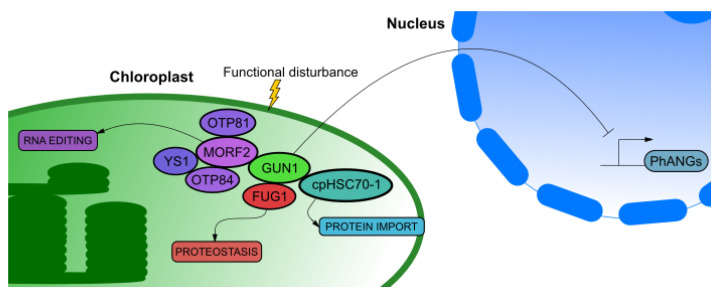
Retrograde signals mediated by GENOMES UNCOUPLED 1 (GUN1). In response to functional disturbance of chloroplast GUN1 inhibits expression of photosynthesis-associated nuclear genes (PhANGs), regulates protein import through interaction with cpHSC70-1, maintains proteostasis through interaction with FUG1 and modulates RNA editing through interaction with MULTIPLE ORGANELLAR RNA EDITING FACTOR 2 (MORF2).

An exception is a GUN1 protein which is not involved in tetrapyrrole synthesis pathway. There are many different hypotheses about exact role of GUN1, but most of them are related to plastid protein homeostasis [84,85,86,87]. When proper functioning of chloroplasts is disturbed GUN1 can also regulate chloroplast RNA editing by physical interaction with MULTIPLE ORGANELLAR RNA EDITING FACTOR 2 (MORF2) to affect maturation of many transcripts among which are subunits of plastid encoded RNA polymerase. MORF2 interacts with ORGANELLE TRANSCRIPT PROCESSING 81 (OTP81), ORGANELLE TRANSCRIPT PROCESSING 84 (OTP84) and YELLOW SEEDLINGS 1 (YS1). *otp81*, *otp84* and *ys1* mutants exhibit weak *gun* phenotype which is enhanced in double and triple mutant in those genes. Overexpression of *MORF2* results in strong *gun* phenotype similar to *gun1* mutant suggesting that plastid RNA editing and retrograde signaling are functionally connected [16]. Another GUN1 interacting protein is FUG1 which functions as translation initiation factor in chloroplasts. Both functional proteins are required to maintain plastid protein homeostasis [88]. GUN1 probably does not affect plastid gene expression per *se*, but it interacts with chaperone cpHSC70-1 in order to regulate nuclear encoded protein import to chloroplast. This allows to maintain protein homeostasis (proteostasis) in the chloroplasts. Mutation in gene encoding *cpHSC70-1* leads to a *gun* phenotype. However, *gun1* mutant grown under normal conditions does not have affected protein import capacity [89]. On the other hand, it was recently demonstrated that GUN1 can directly bind to heme as well as other porphyrins, increases FC1 activity and also limits heme and protochlorophyllide synthesis [90]. Based on that, GUN1 is linked to the tetrapyrroles which are considered to be retrograde signaling molecules [76]. Complex role of GUN1 and its interactors were described in Figure 3. Role of transcription factors connected with GUN pathways is described further in this manuscript in a section dedicated to biogenic control.

An interesting group of metabolites involved in retrograde signaling and mentioned above are carotenoids. They are main scavengers of ^1^O_2_ in chloroplasts and products of their oxidation: β-carotene (β-CAR) and its oxidation product β-cyclocitral (β-CC) can induce changes in NGE [91]. However, transduction of the signals through this pathway depends on METHYLENE BLUE SENSITIVITY 1 (MBS1) PROTEIN. MBS1 is a zinc finger protein located in the nucleus and in the cytosol. Lack of functional protein in *msb1* mutant caused increased susceptibility to ^1^O_2_ generated during high light stress [92]. Increased GFP fluorescence observed in plants expressing MBS1:GFP under native promoter treated with β-CC lead to conclusion that MBS1 is involved in β-CC retrograde-signaling pathway. It is hypothesized that MBS1 can induce expression of singlet oxygen related genes (SORGs) in order to cope with high light intensity stress [93].

Another metabolite linked to retrograde signaling is methylerythritol cyclodiphosphate (MEcPP) [94]. It is a precursor of isoprenoids and its accumulation is corelated with changes in NGE. Increased levels of MEcPP induce unfolded protein response in the ER [95]. Similar to ROS, MEcPP accumulates after abiotic stresses such as wounding or high light [94]. Hydroxymethylbutenyl diphosphate synthase (GcpE) is an enzyme responsible for reducing MEcPP to hydroxymethylbutenyl diphosphate (HMBPP). GcpE is encoded by *CEH1* gene. This enzyme is a redox sensitive protein which could explain MEcPP accumulation during photooxidative stress [96]. Recently, two new photoreceptor phytochrome B (phyB) mutant alleles that are able to revert phenotype of *constitutively expressing HPL* (*ceh1*) mutant were described. *ceh1* mutant was identified in a screen for regulators of stress induced *hydroperoxide lyase* (*HPL*) gene [94]. *ceh1* mutant exhibits a dwarf phenotype, has high concentration of SA and accumulates MEcPP [97,98].

3′-phosphoadenosine 5′-phosphate (PAP) is another metabolite which can function as a retrograde signaling molecule in response to drought and highlight stress by altering expression of *APX2*, *ELIP2 ZAT10* and *DREB2A* genes [99]. Abscisic acid (ABA) is one of hormones playing major role in response to those stresses and it is synthetized in chloroplasts. In *Arabidopsis thaliana* PAP acts as secondary messenger in ABA regulated stomatal closure and germination [100]. PAP is synthetized from 3′-phosphoadenosine 5′-phosphosulfate (PAPS) by sulfotransferases [101]. It was considered to be a byproduct without function in plants however it can alter RNA catabolism in yeast (*Saccharomyces cerevisiae*) by inhibiting two 5′ → 3′ exoribonucleases (XRNs) [102]. PAP degradation in chloroplasts is mediated by inositol polyphosphate 1-phosphatase (SAL1) which function as nucleotide phosphatase [99]. Point mutation as well as T-DNA insertion in *SAL1* gene result in greater drought tolerance which leads to conclusion that SAL1 is a negative regulator of drought tolerance and it is connected with PAP accumulation [103]. Additionally, functional SAL1–PAP pathway is important for biotic stress responses since mutations in *SAL1* gene lead to higher susceptibility to *Pseudomonas syringae* pv. *tomato* DC3000 and *Pectobacterium carotovorum* subsp. *carotovorum* EC1 [104]. SAL1–PAP retrograde-signaling pathway is well conserved in all land plants [105]. It is also interesting that SAL1 is localized in cytosol [106], nucleus [107], chloroplasts [108] and mitochondria [99]. One of most recent reports shows SAL1–PAP retrograde-signaling pathway involvement in iron homeostasis [109].

## 3. Retrograde Signaling in Regulation of Organelles Biogenesis

Retrograde-pathway-transducing signals from plastids to the nucleus in order to regulate chloroplast biogenesis are often called “biogenic control” [110]. Main purpose of this type of retrograde signaling is to modulate NGE so proteins encoded by several of PhANGs can be produced and transported to chloroplasts during their development from proplastids [111]. The coupled expression of PhANGs and PhAPGs allows proper folding and assembly of photosystem complexes [112]. Disturbances in photosystem stoichiometry leads to photoinhibition because amount of energy absorbed from photons exceeds photochemical efficiency of PSII. Photoinhibition eventually leads to ROS formation which can be lethal for developing seedlings.

Signals conditioning proper plastid biogenesis are connected with tetrapyrrole biosynthesis pathway, changes in plastid gene expression (PGE) and activity of the photosynthetic electron transport (PET). ABI4 was discovered during a screen for ABA-insensitive (*abi*) mutants which are able to germinate in presence of ABA [113]. Different *abi4* alleles were also discovered in independent screens for mutants with altered responses to glucose and other sugars [114,115,116,117]. ABI4 is a TF classified to APETALA2/ethylene-responsive factor (AP2/ERF) family. Genome of *Arabidopsis thaliana* encodes 147 members of AP2/ERF family and many members of this family are involved in signaling pathways including responses to abiotic and biotic stresses [118,119]. ABI4 takes part in mitochondrial retrograde signaling regulating expression of *ALTERNATIVE OXIDASE1a* (*AOX*) and chloroplast retrograde signaling [73,120]. Higher expression of nuclear-encoded *RbcS* in *abi4* mutant compared to the wild-type after NF treatment allowed to conclude that ABI4 is involved in chloroplast retrograde signaling [121]. In addition, it was reported that *abi4* mutants were able to rescue expression of *LHCB1.2* after lincomycin (Lin) treatment [73]. Lincomycin is plastid translation inhibitor and similar to NF it is often used to screen for *gun* phonotype. Both of those treatments cause damage to chloroplast resulting in photobleached phenotype and drastically reduced expression of most PhANGs [73,74,122]. Activation of ABI4 depends on phosphorylation by MPK3/MPK6 [123]. Based on that knowledge ABI4 was established as one of key proteins involved in plastid development [71,123,124]. It was considered to be a nuclear target of GUN1-dependent retrograde-signaling pathway [71,73,76]. Although there were also studies in which researchers were not able to observe *gun* phonotype in *abi4* mutant when quantifying expression of *CARBONIC ANHYDRASE1* (*CA1*), *GOLDEN2-LIKE1* (*GLK2*) and *LIGHT HARVESTING CHLOROPHYLL A/B BINDING PROTEIN1.1* (*LHCB1.1*) [125,126,127]. In contrast to *gun1* mutant, crossing *abi4* mutant with *plastid protein import2* (*ppi2*) mutant did not rescue loss of NGE [128]. These results suggest that ABI4 may act independently from GUN1. Recently, an independent study performed on four different alleles of *abi4* did not support a role of ABI4 in biogenic retrograde signaling and researchers were unable to obtain strong or consistent *gun* phonotype in tested *abi4* mutant alleles [129].

It is interesting that this is not first *gun* ‘dismantled’ by this research group. Before, focusing on ABI4 they decided to investigate role of PTM in biogenic retrograde signaling [130]. PTM is a plant homeodomain (PHD) transcription factor bound to chloroplast envelope [71]. It was proposed that PTM can be cleaved off chloroplast membrane after changes in plastid metabolism and its N terminal domain can affect NGE after its accumulation in nucleus [71]. *ABI4* is one of genes which expression is induced by PTM [71]. Treatments with Lin and NF lead to conclusion that *ptm* mutant exhibit *gun* phenotype because it was able to rescue expression of *LHCB* after treatment with these chemicals. Since its discovery, PTM has been included in many models describing biogenic control pathways [112,131,132,133]. PTM was also further described in literature by the same research group which provided the first report as regulator of flowering after exposure to high light and it takes part in integration of the signals during de-etiolation [134,135]. However, observation of *gun* phenotype in *ptm* mutant was still to be confirmed by another research group. Because of potential important role of PTM in retrograde signaling Matthew Terry group decided to further examine its role under NF and Lin treatment. In conducted experiments they were unable to confirm *gun* phenotype after both Lin and NF treatment. Expression of selected PhANGs in *ptm* mutant after treatment were not elevated compared to the wild-type plants. Authors concluded that PTM should be excluded from existing models describing plastid signaling or at least its role in it is not as important as we thought before [130].

There are also other interesting mutants in genes encoding transcription factors that exhibit *gun* or *gun*-like phenotype such as: *hy5* and *glk1glk2* [136,137]. In contrast to the *gun* phenotype, there are also number of mutants called *happy on norflurazon* (*hon*) that can tolerate higher concentration of NF in comparison to the wild-type plants. One of the identified *hon* mutants had mutation in ClpR4 (HON5) subunit of Clp protease complex which localizes in chloroplasts. Another example is a *hon23* mutant, which has mutation in putative chloroplast translation elongation factor, and it clearly shows that these mutations interfere with chloroplast protein homeostasis [138]. It is also worth mentioning that not only mutants in genes encoding transcription factors, but also other proteins such as *cry1* (encoding blue light photoreceptor) or *coe1* (encoding mitochondrial transcription termination factor 4) can exhibit *gun* phenotype and eventually take part in biogenic control [137,139].

## 4. Retrograde Signaling in Stress Response and Acclimation

Retrograde-pathway-transducing signals from plastids to the nucleus in order to cope with environmental stresses and acclimate to them are often called “operational control”. Since plants are in general immobile and unable to avoid many unfavorable environmental conditions, they had to evolve sophisticated mechanisms in order to survive and effectively reproduce.

Chloroplasts are sensors of visible light and crop yield is often corelated with the efficiency of photosynthesis. Second, but equally important metabolic process that provides energy for plant cell is aerobic respiration. This processes however are sensitive to changes in plant growth environment [140]. Abiotic stresses can result in photoinhibition of PSII and inhibition of carbon assimilation enzymes [141]. One of the ultimate responses to severe abiotic and biotic stresses is PCD. Apart from its major contribution during tissue development, PCD is also a mechanism that allows plants to prevent pathogens from reproducing and spreading to uninfected cells. During abiotic stresses PCD promotes dismantling of a limited number of affected cells to prevent severe systemic damage to the whole organism [142]. Cells undergoing PCD exhibit extensive chromatin condensation and developmental PCD can occur only in specific cell types [143]. One of the most interesting regulators of PCD is LESION STIMULATING DISEASE1 (LSD1). LSD1 acts as a transcription regulator and condition dependent scaffold protein [7,144]. LSD1 is negative regulator of cell death and defense responses. Along with its homolog LOL1 (which exhibits an antagonistic function) they cooperate in order to induce adequate response through PCD. Mutation in *lsd1* results in a runaway cell death (RCD) in a light dependent manner [145,146]. It was proved that reduction of the PSII antenna size, thus reduction of light absorption and EEE pressure by crossing *lsd1* mutant with *cao1* mutant caused an increase of NPQ and reversion of the RCD phenotype in *lsd1* [147]. The reduction of plastoquinone (PQ) pool induced by EEE results in burst of ROS which lead to induction of SA and ethylene dependent signaling pathway through EDS1 and PHYTOALEXIN DEFICIENT4 (PAD4) which lie on the same pathway as LSD1 because RCD phenotype of *lsd1* mutant is abolished by inactivation of ROS, SA and ethylene signaling components [6,147,148]. The overexpression of bacterial salicylate hydroxylase (NahG) fused with chloroplast transit peptide from RbcS in *lsd1* mutant background also abolished RCD indicating correlation between *lsd1* RCD and SA accumulation. Mutation in *lsd1* causes uncoupled expression of PhANGs and PhAPGs before induction of RCD; it was hypothesized that LSD1 can act downstream of GUN-mediated pathway. However, *lsd1* treatment with Lin does not rescue expression of *LHCBs* which indicates that it is not a *gun* mutant. Based on that knowledge LSD1 is more likely involved in operational than biogenic control. Uncoupled expression of photosynthesis-associated genes in *lsd1* cause accumulation of singlet oxygen which result in induction of EX1-mediated PCD. *lsd1*;*ex1* double mutant partially reverts RCD phenotype [5]. Based on that knowledge it is suggested that LSD1 plays a role in regulation of PCD and responses towards biotic and abiotic stresses, it also may be an integrator of SA, ROS (including singlet oxygen), ethylene and other hormones (e.g., IAA) mediated pathways [6]. It is also worth mentioning that LSD1, EDS1 and PAD4 are conditional dependent PCD regulators. For example, in optimal laboratory conditions *lsd1* mutant displays deregulation of over 2000 genes while in the suboptimal field conditions it has 62 deregulated genes and only 43 of those genes were commonly deregulated in both of these conditions [9].

In this manuscript, we mainly focused on retrograde pathways related to functioning of chloroplasts. On the other hand, mitochondria also depend on retrograde signaling during their biogenesis and stress responses. However, it needs further investigation whether chloroplasts and mitochondria induce separate signaling pathways or they converge into the same pathways [149]. Knowing that functioning of both is strongly connected through the energy, metabolism and redox status makes second hypothesis a viable one [150,151,152]. However, it is considered that mitochondrial retrograde signaling plays greater role in nonphotosynthetic tissues. To support this hypothesis, it was observed that overexpression of TF ANAC013 in *Arabidopsis thaliana* resulted in enhanced tolerance of chloroplasts during oxidative stress [153]. It is achieved mostly by dissipating excess of reducing equivalents [154,155,156]. Regulators of mitochondrial retrograde signaling are often identified in genetic screens for TFs that can regulate the expression of nuclear genes encoding mitochondrial proteins related to alternative respiration or stress response. Promoters of *AOX1a*, *UPREGULATED BY OXIDATIVE STRESS* (*UPOX*), *NAD(P)H-UBIQUINONE OXIDOREDUCTASE B2* (*NDB2*) and *CYTOCHROME BC1 SYNTHASE1* (At*BCS1*) are often used in such screens. After high light and antimycin A (electron transport chain blocker similar to cyanide) treatment, AtWRKY40 downregulated and AtWRKY63 upregulated expression of *AOX1a*, *UPOX*, *NDB2* and At*BCS1* suggesting their antagonistic function [157]. Interestingly among identified *AOX* regulators there are many components involved in auxin signaling [158]. Another interesting regulator of mitochondrial retrograde signaling is OM66. *OM66* encodes mitochondrial outer membrane protein and its promoter is highly induced by SA in contrary to promoter of *AOX1a* which is responsive to H_2_O2 and rotenone [159]. Because *PATHOGENESIS*-*RELATED1* (*PR1*) is downregulated in *OM66* mutant and *OM66* overexpressing lines have higher content of SA it was proposed that *OM66* is regulated in a SA dependent manner [157]. SA inhibits both cytochrome and alternative respiratory pathways [160]. It also inhibits alpha-ketoglutarate dehydrogenase (α-kGDH) a tricarboxylic acid cycle (TCA) enzyme [161]. This information along with well-established SA interactions with chloroplasts metabolism suggests that SA may be a link between chloroplast and mitochondrial retrograde signaling [162].

Retrograde signals transduced from mitochondria as well as chloroplasts can change NGE in a similar manner. The convergence of those two retrograde-signaling pathways is often linked to CYCLIN DEPENDENT KINASE E1 (CDKE1). It is encoded by *REGULATOR OF ALTERNATIVE OXIDASE1* (*RAO1*) gene. It was first described as an important component of mitochondrial retrograde signaling in response to inhibitors. Functional kinase is needed to regulate *AOX1a* in response to oxidative (H_2_O_2_) and cold stress [163]. It was also demonstrated that CDKE1 regulates expression of *AOX1a* and *Lhcb2.4* in response to photosynthesis inhibitors such as 2,5-dibromo-3-methyl-6-isopropyl-benzoquinone (DBMIB) and 3-(3,4-dichlorophenyl)-1,1-dimethylurea (DCMU). *cdke1*-mutants under high light stress exhibited *gun*-like phenotype [164]. Based on that it was proposed that CDKE1 can integrate retrograde signals from mitochondria and chloroplasts. KIN10 is a subunit of SnRK1 kinase complex conditioning its catalytic activity and it was proposed as an integrator of stress and energy signaling [165,166]. The interaction between KIN10 and CDKE1 in nucleus was also reported [163]. CDKE1 is a part of mediatory complex regulating RNA polymerase II (RNAP II) dependent transcription. Such complexes are considered to integrate stress signals from organelles and initiate proper transcriptional response [167]. Another similarity can be observed in how mitochondrial retrograde signaling is triggered by stress or dysfunction affecting respiratory electron transport chain or TCA [168]. In order to maintain these processes and their energy production, plants need to change their metabolism. That is why proper communication between organelles and nucleus is required [14].

**Figure 4:**
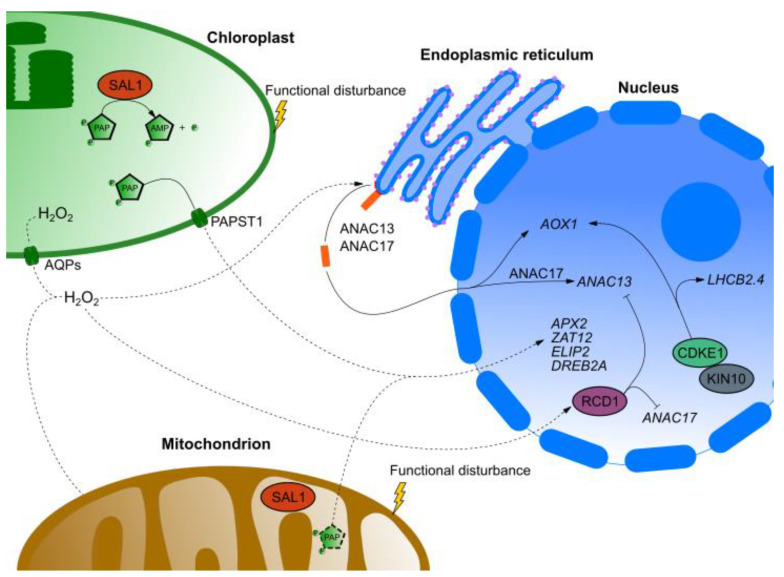
Putative integrators of retrograde-signaling pathways. Inhibition of inositol polyphosphate 1-phosphatase (SAL1) results in accumulation of 3′-phosphoadenosine 5′-phosphate (PAP) which induces expression of many drought and high light related genes such as *APX2*, *ZAT12*, *ELIP2* and *DREB2A*. RCD1 after induction with ROS inhibits expression of *ANAC13* and *ANAC17* which regulates *AOX1* expression. CDKE1 induces expression of *AOX1* and *LHCB2.4* and many other genes as part of mediatory complex regulating RNAPII dependent transcription. Dotted lines represent pathways which lack experimental evidence or exact nature of indicated regulation is still unknown.

Transcriptome meta-analyses demonstrate that 10% to 20% from differentially regulated genes during abiotic stress responses encode proteins localized to the chloroplasts [169]. We briefly described the role of ROS in retrograde signaling in previous sections of this manuscript. However, it is worth mentioning that ROS signaling is crucial to respond to several abiotic stresses such as drought, variable high light intensity, salinity and heat [170]. Exposure to abiotic and biotic stresses can induce unfolding protein response in ER. As was mentioned before it is connected with MEcPP accumulation [171]. Response to drought stress is usually connected to the SAL1–PAP pathway. Some researchers hypothesize that chloroplasts and mitochondrial retrograde signals converge through TF called ANAC017 (encoded by *RAO2*) to regulate PCD as response to severe organellar stress [172]. NAC family members are ER bound TFs which upon activation are cleaved and relocated to nucleus where they can affect NGE [173]. RADICAL-INDUCED CELL DEATH1 (RCD1) is another putative integrator of chloroplastic and mitochondrial ROS signaling pathways. It was identified in a screen for sensitivity to ozone [174]. *rcd1* mutant is resistant to methyl viologen (MV) and UV-B which suggests that RCD1 may be a ROS sensitive protein [175]. In the *rcd1* mutant more than 400 genes are differently expressed under standard growth conditions. Among those genes there are those encoding mitochondrial AOXs as well as chloroplast 2-Cys peroxiredoxin (2CP) [176,177,178,179]. RCD1 interacts with several TFs such as ANAC017 and DREB2A [176,180]. Cleavage of ANAC017 from ER is probably dependent on elevated H_2_O_2_ levels [181]. Recently, RCD1 was proposed to act as negative regulator of ANAC013 and ANAC017 and thus integrator of NAC and PAP retrograde-signaling pathways [182]. Putative integrators of retrograde-signaling pathways and their interactors were demonstrated on Figure 4. Recently, β-cyclocitral induced protein SCARECROW LIKE14 (SCL14) was described along with TF ANAC102 and xenobiotic detoxification enzymes in lowering levels of toxic carbonyls and peroxides in order to limit damage to the intracellular components caused by photooxidative stress [183].

## 5. Conclusions

We briefly demonstrated complexity and different nature of retrograde-signaling pathways. It is commonly considered that in each retrograde pathway there are at least two different types of biomolecules involved. It is worth remembering that the signaling pathways we described are not universal for every cell type and in every epigenetic status. While some of them occur only during development, others such as singlet-oxygen–mediated pathways, can occur only in differentiated, photosynthetically active mesophyll cells. Many existing, even well understood pathways still have unknowns and expanding knowledge about them often brings up more new questions than answers. However, even if we are not yet close to understanding retrograde signaling, benefits from it could be worth the effort. Recently, few interesting strategies to improve crop production under field conditions were demonstrated. First aimed at improvement of photorespiration by implementing synthetic glycolate metabolic pathways into tobacco chloroplasts [184]. Others improved crop production and water-use efficiency by accelerating recovery from photoprotection. It was achieved by combined overexpression of *PsbS* and genes encoding xanthophyll cycle enzymes [185,186]. In the future studies we shall also consider other physical retrograde signaling pathways, for example, direct heat radiation and vibration of organelles, electrical and calcium wave signaling from chloroplasts and mitochondria.

## Abbreviations

^1^O_2_	Singlet oxygen
ABA	Abscisic acid
ABI4	ABSCISIC ACID-INSENSITIVE4
*AOX*	*Alternative oxidase 1a* gene
APX	Ascorbate peroxidase
AQPs	Aquaporins
CAT	Catalase
CDKE1	CYCLIN DEPENDENT KINASE E1
DAD	DEFENDER AGAINST CELL DEATH
EDS1	ENHANCED DISEASE SUSCEPTIBILITY1
EEE	Excess excitation energy
EMS	Ethyl methanesulfonate
ER	Endoplasmic reticulum
EX1	EXECUTER1
EX2	EXECUTER2
FC1	Ferrochelatase 1
GcpE	Hydroxymethylbutenyl diphosphate synthase
GPX	Glutathione peroxidase
GSH	Glutathione
GUN	Genomes uncoupled
H_2_O_2_	Hydrogen peroxide
IAA	Indole-3-acetic acid
ICS1	ISOCHORISMATE SYNTHASE1
JA	Jasmonic acid
*LHCB*	*LIGHT HARVESTING CHLOROPHYLL A/B BINDING PROTEIN* gene
Lin	Lincomycin
LSD1	LESION STIMULATING DISEASE1
MAPK	Mitogen-activated protein kinase
MBS1	METHYLENE BLUE SENSITIVITY 1
MEcPP	Methylerythritol cyclodiphosphate
MgProto	Mg–protoporphyrin
MORF2	Multiple organellar RNA editing factor 2
NF	Norflurazon
NGE	Nuclear gene expression
NOXs	NADPH oxidases
NPQ	Nonphotochemical quenching
O_2_•-	Superoxide anion radical
OGE	Organellar gene expression
OH●	Hydroxyl radical
OXI1	Oxidative signal inducible 1
PAD4	Phytoalexin deficient4
PAP	3′-phosphoadenosine 5′-phosphate
PCD	Programmed cell death
PET	Photosynthetic electron transport
PGE	Plastid gene expression
PhANGs	Photosynthesis-associated nuclear genes
PhAPGs	Photosynthesis-associated plastid genes
PrxR	Peroxiredoxin
PSI	Photosystem I
PSII	Photosystem II
PTM	Plant homeodomain transcription factor with transmembrane domains
*RbcS*	*RIBULOSE-1,5-BISPHOSPHATE CARBOXYLASE/OXYGENASE SMALL SUBUNIT* gene
RBOH	Respiratory burst oxidase homologs
RCD1	Radical-induced cell death1
RNAP II	RNA polymerase II
ROS	Reactive oxygen species
SA	Salicylic acid
SAFE1	Safeguard1
SAL1	Inositol polyphosphate 1-phosphatase
SOD	Superoxide dismutase
SORGs	Singlet oxygen related genes
TBP	Tetrapyrrole biosynthesis pathway
TCA	Tricarboxylic acid cycle
TF	Transcription factor
β-CAR	β-carotene
β-CC	β-cyclocitral
